# Gender-Based Comorbidity in Benign Paroxysmal Positional Vertigo

**DOI:** 10.1371/journal.pone.0105546

**Published:** 2014-09-04

**Authors:** Oluwaseye Ayoola Ogun, Kristen L. Janky, Edward S. Cohn, Bela Büki, Yunxia Wang Lundberg

**Affiliations:** 1 Boys Town National Research Hospital, Omaha, Nebraska, United States of America; 2 Department of Otolaryngology, Karl Landsteiner University Hospital Krems, Krems an der Donau, Austria; University of Iowa Carver College of Medicine, United States of America

## Abstract

It has been noted that benign paroxysmal positional vertigo (BPPV) may be associated with certain disorders and medical procedures. However, most studies to date were done in Europe, and epidemiological data on the United States (US) population are scarce. Gender-based information is even rarer. Furthermore, it is difficult to assess the relative prevalence of each type of association based solely on literature data, because different comorbidities were reported by various groups from different countries using different patient populations and possibly different inclusion/exclusion criteria. In this study, we surveyed and analyzed a large adult BPPV population (n = 1,360 surveyed, 227 completed, most of which were recurrent BPPV cases) from Omaha, NE, US, and its vicinity, all diagnosed at Boys Town National Research Hospital (BTNRH) over the past decade using established and consistent diagnostic criteria. In addition, we performed a retrospective analysis of patients’ diagnostic records (n = 1,377, with 1,360 adults and 17 children). The following comorbidities were found to be significantly more prevalent in the BPPV population when compared to the age- and gender-matched general population: ear/hearing problems, head injury, thyroid problems, allergies, high cholesterol, headaches, and numbness/paralysis. There were gender differences in the comorbidities. In addition, familial predisposition was fairly common among the participants. Thus, the data confirm some previously reported comorbidities, identify new ones (hearing loss, thyroid problems, high cholesterol, and numbness/paralysis), and suggest possible predisposing and triggering factors and events for BPPV.

## Introduction

The peripheral vestibular organ consists of miniature inertial accelerometers: the semicircular canals (SCCs) specialized in detecting angular acceleration, and the otolithic organs in sensing linear acceleration including gravitational changes. In benign paroxysmal positional vertigo (BPPV), according to the accepted theory, utricular otoconia break apart from the mass and become dislocated into the semicircular canals. Thereby, the canals are sensitized to gravity and generate false information of angular acceleration in response to head position changes with respect to gravity.

BPPV is the most common cause of vertigo. In young people, one third to one half of BPPV cases can be attributed to some type of head trauma/injury [1]–[5]. A number of surgery-induced BPPV cases, especially inner ear and dental surgeries, have also been reported [6]–[10]. In the absence of head trauma or mechanical impact (e.g. surgical drilling) to cause otoconia dislocation, BPPV is categorized as unknown origin or idiopathic. In middle aged and older people, idiopathic BPPV cases are much more common. It is becoming increasingly evident that idiopathic BPPV cases can be associated with other illnesses such as migraine [9], [11]–[16], vestibular neuritis [17], [18], Meniere’s disease [9], [19]–[21], sudden hearing loss [19], [22]–[27], diabetes [28], [29], and autoimmune thyroiditis [31]. BPPV has also been linked with reduced bone mineral density [32]–[34], suggesting that the spontaneous release of otoconia may parallel bone demineralization (for review see [30]).

Unfortunately, the methods of the reported epidemiological studies were not standardized, and possibly used different diagnostic and inclusion/exclusion criteria. Sometimes there is disagreement between reports whether BPPV is associated with some diseases/conditions. For example, Warninghoff et al [35] did not detect an increased prevalence of diabetes mellitus, arterial hypertension, migraine, other headaches or obesity in the BPPV patients compared to the control population, likely due to a small sample size (n = 19 BPPV patients). Given some inconsistencies in the comorbidity findings and the scarcity of epidemiological studies in the US, we surveyed and reviewed the medical records of a large BPPV patient population diagnosed and treated at BTNRH in the current study, and identified predisposing and triggering factors and events. Our long-term goal is to use the information as a starting point to study the pathophysiology of BPPV, as well as help improve or modify treatment and prevention strategies.

## Materials and Methods

### Study design and subjects

Diagnostic records of BPPV patients at the BTNRH Vestibular Clinic between 2002–2011 (n = 1,377, with 1360 adults and 17 children) were analyzed after patients’ information was de-identified and anonymized. The quoted numbers of patients in this study excluded a small subset that also had other types of vertigo/dizziness in addition to BPPV. Then, adult patients (n = 1,360) were directly surveyed using an anonymous questionnaire. Only the participants who completed the questionnaire in its entirety with evaluable data were included. A positive diagnosis of BPPV was the presence of nystagmus elicited in either the Dix-Hallpike or roll maneuver. The current study did not differentiate or exclude sub-types of BPPV, such as subjective BPPV [36] or BPPV sub-typed by location (posterior, lateral or superior canal) [37]. Our purpose was to investigate comorbidities that predispose an individual to freeing otoconia and not location of otoconia displacement; sub-typing would result in very small numbers in some sub-groups for statistical analysis.

All aspects of the study, including the retrospective analysis, the content of the survey, the invitation letter and the survey procedures, were reviewed and approved by the Institutional Review Board at BTNRH in accordance with institutional, federal and international guidelines (approval number 11-11-X). As described above, patients’ information was de-identified and anonymized prior to analysis. Since the retrospective analysis and the adult-only survey were done anonymously, no consent was necessary.

### Procedures

Initially, a retrospective review of the medical records of the stated BPPV patient population was conducted, which provided preliminary information on comorbidities of BPPV and the rate of BPPV recurrence.

Then, a survey was created and administered through Survey Monkey (www.surveymonkey.com). Most questions required the participant to select one answer from two to several choices, with additional lines to add other information or explanation by the participant. When applicable, the choices included “I am not sure”, “I cannot remember” or “Does not apply”. The survey was divided into the following sub-sections:

1. Demographic information, including age (in years), sex, race, ethnicity, and general area of residence (urban/suburban or rural).
2. BPPV recurrence, family history, season of onset. Recurrence was defined in the survey as experiencing an additional episode of BPPV after being symptom-free for at least 30 days.
3. Events/illnesses immediately preceding BPPV onset, medical history, medications and diet. A table (see Results) contained a list of events/illnesses and asked whether the participant experienced them prior to BPPV. The “prior to” was arbitrarily defined as “less than 6 months before”. With the exception of head trauma, we speculated that other events/illnesses would not immediately result in BPPV, so we were looking for associations in a more loose time-locked fashion. The survey questions were asked as listed in the tables in the Results, except that the phrase “Did you experience xxx prior to your first BPPV symptom (or during your BPPV symptoms)” was included in each line item for each condition/disease in the survey. In the table, this phrase is summarized in the legend to save space.

In the medical history section, participants were given a comprehensive list of medical conditions to select from and blank lines to fill in any other medical conditions. Participants were asked to list their current medications, and to indicate whether their medical conditions and medication taken co-occurred with BPPV onset. Some questions regarding events/illnesses immediately preceding BPPV onset somewhat overlapped questions in the medical history section, thus serving as confirmation.

Frequency of consumption of alcohol, tobacco, caffeine, tea or carbonated beverages, high fat/sugary/salty foods, fresh fruits/vegetables, and meat/fish was filed as never, less than once a week, 2–3 times a week, or more than 3 times a week for each stated item. Consumption of special diet (e.g. vegetarian) was also asked.

An invitation letter to participate in the study was mailed to 1,360 adult BPPV patients. Participants had the option of completing the survey online or on paper. Some surveys were also distributed to new patients at the time when BPPV was diagnosed at BTNRH. All responses were downloaded into Excel, which served as the database for review and analysis.

For comparison, age- and gender-matched prevalence data on the general US population were obtained from the Center for Disease Control (CDC), the National Institute of Health (NIH), and peer-reviewed published papers. With a few diseases, control data on the general US population stratified by age and gender were not available, so European gender-based data on similar age groups were used. Such cases are indicated in the tables in the Results section.

### Statistical analysis

The total number of participants who answered each question and the subtotals who gave one type of answer were tallied. When calculating the percentage of each subtotal over the total, those who answered “I am not sure”, “I cannot remember” or “It does not apply” were not included in the total or subtotal. The control statistics usually consisted of thousands to hundreds of thousands of people. To provide an estimate of the statistical significance of the difference in the disease prevalence between BPPV patients and the general population, p values were calculated for each comorbid disease using Fisher’s exact test. To simplify the analysis and to provide a conservative estimate of statistical significance, the control population size was set at 1,000 for all comorbidities, and the mean values from meta-analysis (if available) or the highest prevalence data available were used in the analysis.

The ages of the survey participants (n = 227) and the entire adult BPPV population (n = 1,360) were compared using Student’s t-test, and statistical significance of the effects of comorbid conditions on BPPV recurrence was evaluated using Fisher’s exact test. Familial cases of BPPV (a 2^nd^, 3^rd^ and more cases of BPPV in blood relatives in a family) were compared with the highest reported prevalence (life-time prevalence) of BPPV in the general population using Fisher’s exact test.

## Results

### Demographics

**Figure 1:**
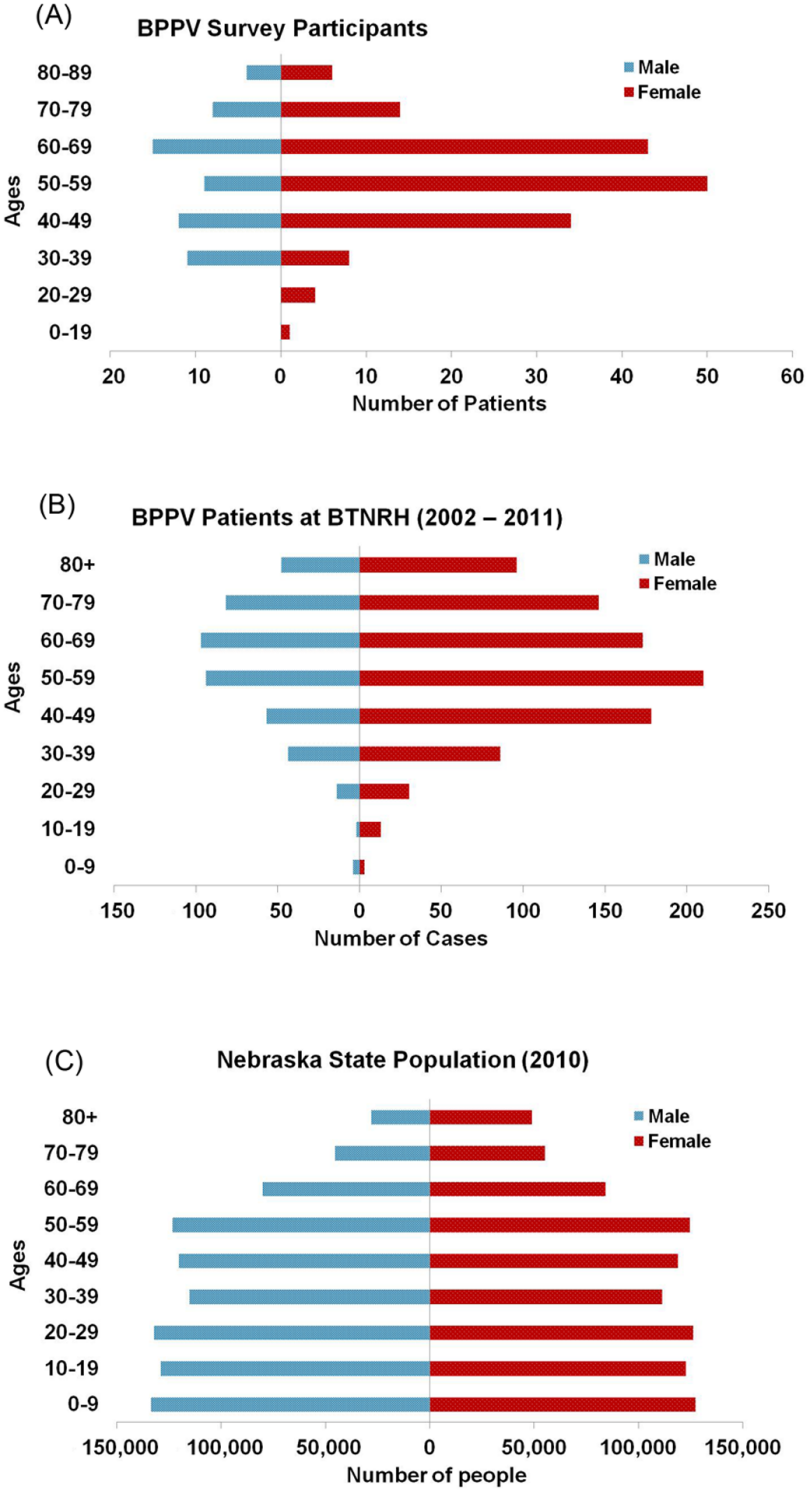
Age and gender distribution of BPPV survey participants (n = 227 total) (A), BPPV cases diagnosed at BTNRH in 2002–2011 (n = 1,377 total) (B), and Nebraska population in year 2010 (n = 1,826,341 total) [73] (C). Recurrent cases are counted only once (the first occurrence).

Of the 1,360 total adult patients, 227 (164∶63 or 2.6∶1 female to male) completed the survey in its entirety. Characteristics of the two groups are summarized in **Table 1**. The survey participants had an age and gender distribution similar to the entire patient population under invitation (**Figure 1**), except that the former had slightly fewer people above 70 years old.

**Table 1:** Demographics of the study population.

	BPPV adult patient population			BPPV survey participants			
	N	Age range(years)	Mean age(years)	N	Age range(years)	Mean age(years)	P
**Female**	923	18–94	58.3±16.0	164	19–84	56.1±13.0	0.04
**Male**	437	18–91	60.2±15.7	63	30–86	56.4±15.2	0.0007
**Gender mixed**	1,360	18–94	58.9±15.9	227	19–86	56.2±13.6	0.006

Among the survey participants, the ethnicity distribution was 80.9% “Not Hispanic or Not Latino”, and 19.1% “Hispanic or Latino”. The racial distribution was 95.8% White, 2.5% African American or Black, 1.3% Asian and 0.4% American Indian or Alaskan. Of the participants, 69.9% live in urban and suburban areas, 28.0% live in rural areas and 2.1% were not sure.

### Recurrence of BPPV

In the BPPV survey population (n = 227), 174 participants (131 females and 43 males) reported recurrence of BPPV (53 non-recurrent cases), resulting in a recurrence rate of 76.3%. This is higher than the recurrence rates (27%–50%) reported by others [38], [39], [40], suggesting that recurrence may have been a strong motivation for participation.

**Figure 2:**
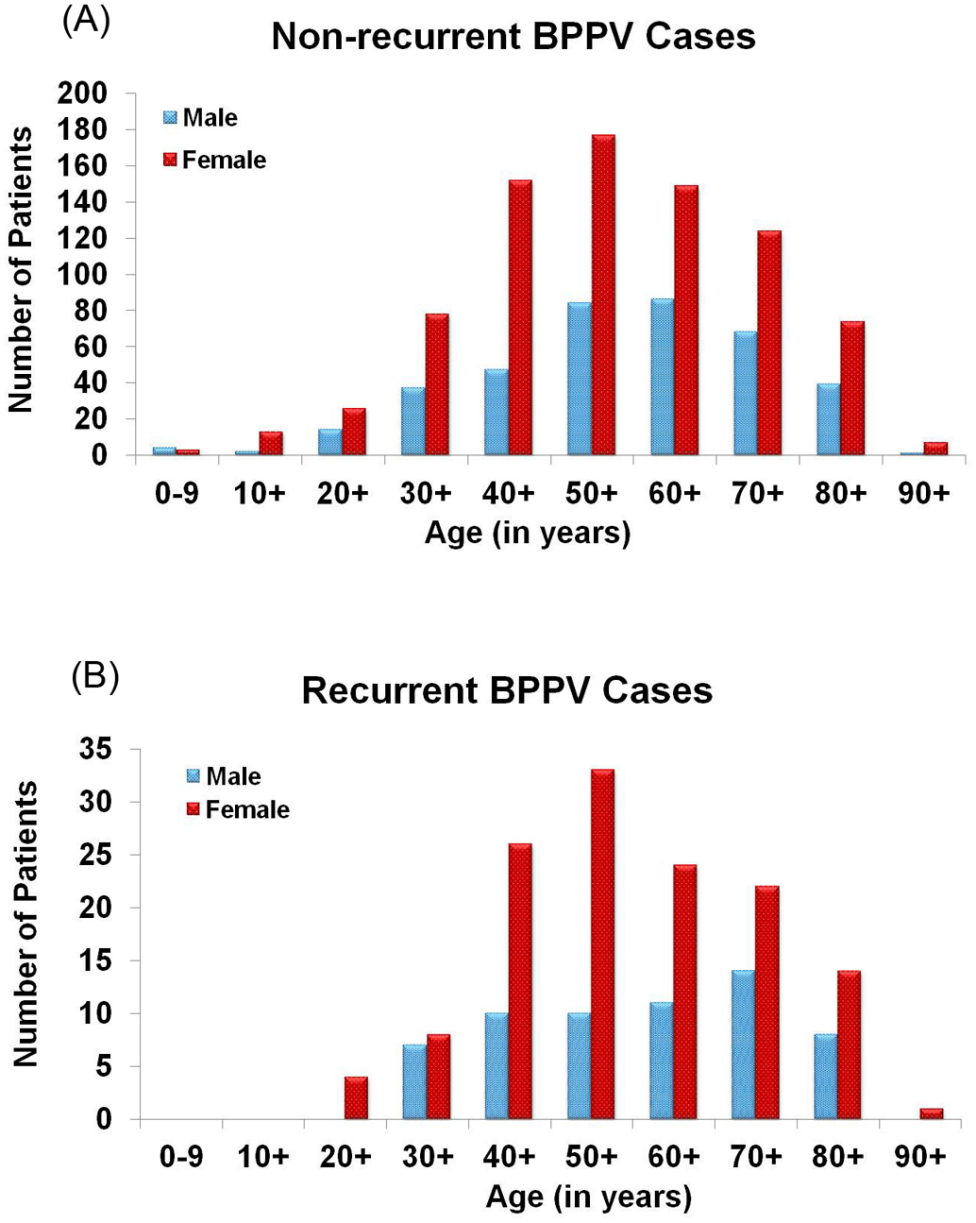
Age and gender distribution of non-recurrent (A, n = 1,185 total) and recurrent (B, 192 total) BPPV cases diagnosed at BTNRH from 2002–11.

Among the entire BPPV population from 2002–2011 (n = 1,377), female recurrent and non-recurrent BPPV cases had a similar age distribution (**Figure** **2**), but male recurrent cases had an even greater age-related increase in the 70s group when compared to male non-recurrent cases. There were no recurrent female cases in the first 2 decades of life, and no recurrent male cases in the first 3 decades of life. In terms of recurrence, there was no significant difference between urban and rural dwellers, or in the frequencies of different types of diets or vitamin use. It could not be determined in the current study whether medication could have been a factor in BPPV occurrence or recurrence.

### Family history and other information

Among the survey participants, 23.8% (n = 54) reported having a known family member diagnosed with BPPV, 64.8% (n = 147) reported not having a family member with BPPV, and 11.4% (n = 26) did not know if any family member had BPPV. The affected family members were all blood-related except for 3 cases of non-genetic family members (spouses) who also had BPPV (**Table 2**). The frequency of having a blood relative diagnosed with BPPV is significantly higher (p<0.0001) than the 2.4% life-time prevalence reported for the general population [41].

**Table 2:** Family history of BPPV.

Family member	% of yes (n = 54 total)
Parents	29.6% (n = 16)
Children	14.8% (n = 9)
Siblings	27.8% (n = 16)
Siblings and children	1.9% (n = 1)
Parents & siblings	11.1% (n = 6)
Grandparents/uncles/aunts/cousins	7.4% (n = 4)
Spouse	5.6% (n = 3)
No response	1.9% (n = 1)

Among the 68.4% (n = 227) of survey participants who remembered with certainty the season of BPPV onset, a higher percentage of participants had BPPV symptoms in the spring and fall: 28.2% in the spring, 18.0% in the summer, 28.9% in the fall, and 24.9% in the winter. Medical records showed that in the entire BPPV population, April and August had the highest number of cases at 11.0% and 10.7%, respectively. The higher incidences in the spring and fall implicate allergy in facilitating BPPV onset, which is supported by patients’ self-reported problems with allergy (see below).

### Possible triggers and comorbidities of BPPV

When asked about incidents and conditions immediately preceding BPPV symptoms, most participants reported hearing loss (**Table 3**). The next most common problem was headache associated with light and/or sound sensitivity and migraine, followed by infection. Most common infection was ear infection (28.6%, n = 10), sinus infection (28.6%, n = 10), and both (17.1%, n = 6).

**Table 3:** Incidents and conditions immediately preceding the first BPPV symptoms.

Answers	Yes (n, %)	No (n, %)
**Hearing loss**	**93 (41.9%)**	129 (58.1%)
Any other ear problems	14 (6.2%)	211 (93.8%)
Current ear problems	55 (24.3%)	171 (75.7%)
Tinnitus	6 (4.0%)	144 (96.0%)
Headaches associated withincreased light/sound sensitivity	47 (20.9%)	178 (79.1%)
Migraine	28 (12.4%)	197 (87.6%)
Any infection	37 (16.5%)	187 (83.5%)
Any illness	32 (14.3%)	192 (85.7%)
Head injury	27 (12.0%)	198 (88.0%)
Other physical trauma	13 (5.8%)	212 (94.2%)
Any ear surgery	13 (5.8%)	212 (94.2%)
Any other surgery	32 (14.3%)	192 (85.7%)
Any other symptoms	60 (27.0%)	162 (73.0%)

Only those who answered “yes” or “no” were included in the total or subtotal to obtain the percentages. Those who answered “does not apply” or “do not know/remember” were not included in the presented percentages.

In the Medical History section, the comprehensive list provided information on possible comorbidities and whether they co-occurred with BPPV, and confirmed those of the previous section in the survey (**Tables 4** **&** **5**). Some differences in the answers may not be inconsistencies but may be the result of how the questions are phrased: one seeks information about the incidents/illnesses immediately preceding the first BPPV symptoms (**Table 3**), while the second seeks information about their chronic situations that may or may not accompany the vertigo attacks (**Tables 4** **&** **5**). Given the gender differences in comorbidities, male and female data were analyzed separately.

**Table 4:** Factors associated with BPPV in females.

Comorbidities	Female BPPV patientsyes% (n out of 164 total)	General female population(set at n = 1,000)	P	References and notes for control data
**Allergies**	50% (82)	32.3%–35% (350)	**0.0003**	[56], [57]
Blood pressure	38.4% (63)	26.4–32.8% (328)	0.18	[58]
**Ear/Hearing**	33.5% (55)	12.7–14.4% (144)	**<0.0001**	[58] Estimated based on gender ratio and data in 45–64 age group
**Head injury**	8.5% (14)	3.0% (30)	**0.003**	[59] Annual occurrence (0.3%) x 10 years
**Headaches**	38.4% (63)	17.0–38.0% (275)	**0.005**	[60], [61] Mean from meta-analysis; Chronic or repeated headaches were assumed
**Heart**	17.7% (29)	12.0% (120)	**0.058**	[58] Estimated from gender ratio
**High cholesterol**	39.0% (64)	31.0% (310)	**0.047**	[62]
Kidney/bladder	10.4% (17)	6.6% (66)	0.10	[58], [63] 1.8% chronic kidney diseases + 4.8% chronic bladder problems
**Migraine**	26.2% (43)	19.7% (197)	**0.06**	[64]
Numbness/Paralysis	9.2% (15)	5.6% (56)	0.11	[65], [66] Control on numbness (3.8%) only had stroke data (including mild stroke); on paralysis (1.8%) included injury cases
**Thyroid**	21.3% (35)	13.2% (132)	**0.008**	[67]–[69] Data on UK population

The age- and gender-matched control statistics are on the general population in the size of thousands to hundreds of thousands. To simplify the analysis and to provide a conservative estimate of statistical significance, the control population size is set at 1,000 for all diseases for Fisher’s exact test in the table. If no mean prevalence data by meta-analysis are available, the highest prevalence data available for each disease in the control population is used to obtain the two-tailed p values. Significant (or nearly significant) comorbidities are bolded.

**Table 5:** Factors associated with BPPV in males.

Comorbidities	Male BPPV patientsyes% (n out of 63 total)	General male population(set at n = 1,000 total)	P	References and notes for control data
Allergies	38.1% (24)	27.6% (276)	0.08	[57] Control are non-US population
Blood pressure	44.4% (28)	33.9% (339)	0.10	[58]
Cancer	14.3% (9)	9.5% (95)	0.27	[58] Estimated based on gender ratio and data in 45–64 age group
**Ear/Hearing**	47.6% (30)	23.4% (234)	**<0.0001**	[58] Estimated based on gender ratio and data in 45–64 age group.
**Head injury**	25.4% (16)	4.0% (40)	**<0.0001**	[59] Annual occurrence (0.4%) x 10 years
Headaches	20.6% (13)	12.5–31.0% (218)	1.00	[60] Mean from meta-analysis; Chronic or repeated headaches were assumed
**High cholesterol**	50.8% (32)	32.5% (325)	**0.004**	[62]
Kidney/bladder	12.7% (8)	6.8% (68)	0.12	[58], [63], [70], [71] 2.0% chronic kidney diseases + 4.8% chronic bladder problems
Migraine	11.1% (7)	7.0% (70)	0.21	[64]
**Numbness/Paralysis**	14.3% (9)	5.1% (51)	**0.007**	[65], [66] Control on numbness (3.0%) only had stroke data (including mild stroke); on paralysis (2.1%) included injury cases
Seizures	4.8% (3)	1.7% (17)	0.11	[72]
**Thyroid**	11.1% (7)	1.5% (15)	**0.0002**	[67]–[69] Data on UK population

See Table 4 legend.

Among the 3 dozen listed categories of diseases and medical conditions, about a dozen had a different prevalence than that of the general population, with only some reaching significance (**Tables 4** **&** **5**). Problems with the ear/hearing were again among the most common complaints in both genders. Incidences of headaches/migraine and head injury were also consistent with the previous question, and were common in females and males (especially young males), respectively. With male participants, the listed decades of life (1–9) had 0%, 0%, 0%, 45.5%, 23.1%, 36.4%, 21.4%, 0%, 25%, respectively, of head injury cases. With females, each decade had 0%, 0%, 1.7%, 20.0%, 6.0%, 5.4%, 15.8%, 0%, 0%, respectively, of head injury cases. When all age groups and genders were combined, the percentage of BPPV cases with head trauma was 12.4%, comparable to the reported range of 14%–18% in other BPPV populations [4], [29], [42], [43]. Because it has not been established that head and physical trauma in these patients were actually the cause of their BPPV (e.g. someone may have had head trauma and hearing loss at the same time), these participants were not excluded in the analysis of other comorbidities.

In both genders, problems with thyroid and high cholesterol were significantly more prevalent in the BPPV population (**Tables 4** **&** **5**), although the comparison for thyroid diseases was made with control data from a large study conducted in the United Kingdom (UK) as age- and gender-matched data were not available on the US population.

In the BPPV female population, complaints of allergies and headaches were significantly more prevalent than in the control population. The former is consistent with above mentioned trend of BPPV occurring more frequently during allergy season. Musculoskeletal disorders (28.3%) were not more common in BPPV patients than the general population; however, it is unclear if the survey participants have had a bone scan, therefore, a negative answer to the question whether they had osteoporosis/osteopenia may not exclude the presence of the condition in some people.

Among the conditions that had a significantly higher prevalence in the BPPV population, some also appeared to increase disease recurrence (**Table 6**); however, only numbness/paralysis reached statistical significance. Neither ear surgery nor head injury impacted BPPV recurrence significantly. Gender-separated analysis did not significantly improve the p values; it increased the p values with most of the comorbidities.

**Table 6:** Effects of comorbid conditions on BPPV recurrence.

Comorbidities	RR	OR	P	N
Allergies	0.94	0.77	0.43	76/27∶91/25
Ear/Hearing problems	1.12	1.64	0.15	74/17∶93/35
High cholesterol	1.10	1.54	0.20	75/18∶92/34
Headaches	0.98	0.93	0.87	55/18∶112/34
Migraine	1.08	1.41	0.44	38/9∶129/43
**Numbness/Paralysis**	1.29	7.34	**0.03**	21/1∶146/51
Thyroid disease	1.14	1.96	0.22	34/6∶133/46
Physical trauma	1.23	3.95	0.30	12/1∶155/51

The relative risk (RR) and odds ratio (OR) of BPPV recurrence in the presence vs. absence of a comorbid condition were calculated. The actual case numbers (N) are also presented in the order of recurrent/non-recurrent cases with and without the comorbid condition. Fisher’s exact test was used to obtain the two-tailed p values.

In the medical records of BPPV cases in 2002–2011 (n = 1,377), there were 67 cases of hearing loss (60 sensorineural hearing loss, 6 conductive, 1 unspecified sudden hearing loss), 20 hypoactive labyrinth, 10 migraine, 8 Meniere’s disease, 7 allergic rhinitis, 5 infective otitis externa, and 2 viral labyrinthitis. These comorbidities were reportedly co-occurring with BPPV at the time of the clinical visits, making them appear less prevalent than hearing loss reported in the survey. It is also possible that many patients did not mention their other medical conditions as vertigo was their primary concern during the clinical visits, or other diagnoses were not recorded.

## Discussion

Due to the high prevalence and potentially incapacitating nature of BPPV, the present study examined factors that may predispose an individual to BPPV. We found a few significant comorbidities and familial predisposition for BPPV.

Notably, the number of cases with hearing loss is quite high among our BPPV patients, whereas previously reported numbers were quite low (less than 1%) [19], [44] except for incidences of sudden hearing loss. Sudden hearing loss has commonly been reported in BPPV patients, ranging from 9–51% [24]–[27], [45]; however, sudden hearing loss was rare among the BTNRH BPPV cases in the medical records (n = 1,377). Given that most of our BPPV patients are beyond middle age, we postulate that their hearing loss associated with BPPV is more likely caused by age-related degeneration, rather than by immune or inflammatory responses proposed as the cause of some cases of sudden hearing loss. This hypothesis would be in agreement with the observed effects of aging on BPPV in this population [46] and in other BPPV populations as well (for references see Introduction).

Nevertheless, our survey suggests that ear infection and inflammatory processes (infection and allergy) may also trigger BPPV. Increased incidences of sinus infections in other BPPV populations in the US have been noted [47]. Nasal allergies have also been associated with increased inner ear pathology [48]. The data suggest a possible association between allergies and BPPV onset, especially in women. Although our survey does not specify the type of allergies, nasal allergy is the most common type of allergies in the general population.

We also identified thyroid problems as a possible predisposing factor for BPPV occurrence. Thyroid problems disrupt the homeostasis of calcium and chloride [49], both of which affect otoconia in animal studies [50]–[52]. In addition, hypothyroidism delays the onset of inhibitory neurotransmission [49] during development. Although not yet examined in adults, if hypothyroidism also negatively affects inhibitory transmission in adulthood, it can lead to an imbalance of excitatory/inhibitory transmission. Indeed, autoimmune thyroiditis has been associated with BPPV [19], [30], [31].

Other previously unknown factors that we identified to be associated with BPPV include high cholesterol and numbness/paralysis. It is not clear how these and other factors in Tables 4 & 5 could be biologically linked to BPPV, but immobility may be a factor. For example, there are reports of BPPV possibly arising from bed-rest after surgeries which do not have mechanic impact on the ear or head [53].

In a concurrent study [46], we found that menopause is a major trigger of BPPV. Another common menopausal disease is osteoporosis and osteopenia. Although our data do not provide a definitive conclusion regarding the association of BPPV with osteoporosis or osteopenia, several other independent groups from different countries have reported significantly reduced bone mineral density and increased incidences of osteoporosis/osteopenia in BPPV patients [32]–[34]. Many other diseases can also be triggered or aggravated by menopause, such as allergies, high blood pressure, high cholesterol, headaches, migraine, heart diseases, and hearing loss [54], whether or not the condition shows a female preponderance in the overall prevalence. Most of these conditions showed a significantly higher prevalence in the BPPV patients than the age- and gender- matched controls (Table 4). Our survey data showed a possible association between migraine and BPPV when compared to the general population, and a strong association between generalized headache and BPPV. It is not clear if the higher migraine prevalence in BPPV patients reported by other investigators [16]; [12] reached statistics significance. Ishiyama et al. [12] noted a younger average age of onset and a higher recurrence rate of BPPV in patients with migraine than those without. Our migraine prevalence in BPPV patients may have been underestimated, because our survey invitation letter and an earlier part of the questionnaire emphasized a disease as “diagnosed”, so the participants likely only selected “yes” to migraine if they have been diagnosed. Also, BTNRH does not have a neurology specialty, and some patients with migraine may ignore their BPPV symptoms or not come to BTNRH clinics.

Our data confirm the previously noted familial predisposition in BPPV occurrence [55]. With the exception of 3 spousal cases, the affected family members are all genetic relatives. Anecdotally, vestibular clinicians have encountered twins who have lived apart but all have suffered BPPV (Janet O Helminski, personal communication). All these facts implicate a genetic contribution, not similar life styles or environment, in causing these familial BPPV incidences.

The survey response rate of 16% (18.5% when incomplete responses are counted) is reasonable because we had a long questionnaire, and the invitation letter was mailed on paper rather than emailed with a link (email is more convenient and could improve the response rate). As shown in Figure 1, the response is good representation of the entire BPPV population at BTNRH except for those above 70 years of age. Although comorbid diseases in very old patients may be missed, our findings may be more specific to BPPV rather than as a non-specific coincidence of aging. It should be noted that the majority of the participants were recurrent BPPV sufferers, therefore, the observed comorbidities may be somewhat biased toward BPPV recurrence even though most of the effects did not reach statistical significance (Table 6). Another potential bias of the study is that BTNRH is primarily an otolaryngology and pediatrics facility, and the patient data may over- or under-represent some comorbidities, such as hearing loss (possibly over), migraine (under), and high blood pressure (under). We anticipate such possibility to be small because these clinical visits at BTNRH were sought by patients for BPPV symptoms. Furthermore, certain conditions that showed a significantly higher prevalence in BPPV patients were also reported to occur shortly before the onset of BPPV (Table 3). When BPPV is consistently associated with certain diseases in studies done by independent groups using different patient populations, it would be important to conduct future studies to examine the biological mechanism and genetic predisposition to explain the link between these comorbidities and BPPV.

## Conclusion

Our data show a significant effect of gender, familial predisposition, and certain conditions/diseases in BPPV etiology. Some of these associations (hearing loss, thyroid problems, high cholesterol, and numbness/paralysis) were previously unknown or are much higher than previously reported. When other investigators’ findings are also considered, it may be beneficial to the patients if clinicians diagnosing BPPV are alerted about potential comorbid conditions and consider screening these patients or checking their medical history for the following conditions: hearing loss, head injury, thyroid, lipid, allergies, headaches, diabetes, bone density, and family history.
